# Dissociating the Electrophysiological Correlates between Item Retrieval and Associative Retrieval in Associative Recognition: From the Perspective of Directed Forgetting

**DOI:** 10.3389/fpsyg.2016.01754

**Published:** 2016-11-07

**Authors:** Yujuan Wang, Xinrui Mao, Bingbing Li, Wei Wang, Chunyan Guo

**Affiliations:** ^1^Beijing Key Laboratory of Learning and Cognition, Department of Psychology, Capital Normal UniversityBeijing, China; ^2^Beijing Advanced Innovation Center for Imaging Technology, Capital Normal UniversityBeijing, China

**Keywords:** item retrieval, associative retrieval, FN400, LPC, directed forgetting

## Abstract

Although many behavioral studies have reported associative memory was different from item memory, evidence coming from ERP researches has been in debate. In addition, directed forgetting effect for items has been fully discussed, but whether association between items can be directed-forgotten was unclear. The directed forgetting effect was important for dissociating the item retrieval and associative retrieval because of the one-to-one mapping relationship both between item retrieval and familiarity and between associative retrieval and recollection. Thus, the aim of this study was to investigate the dissociation between item retrieval and associative retrieval and test directed forgetting effect for associative information. Associative recognition paradigm combined with directed forgetting paradigm by ERP recording was employed. Old/rearranged effect in to-be-remembered condition, which was associated with associative memory, was significant at 500–800 ms (LPC) but not at 300–500 ms interval (FN400), indicating that item information was retrieved prior to associative information. The ERP wave calculated by subtracting the to-be-forgotten old pairs with “old” response from those with “rearranged” response, which reflected associative retrieval in the to-be-forgotten condition, was negative from 500 to 800 ms (reversed old/new effect), indicating that association between items can be directed-forgotten. Similar evidence was obtained by contrasting “rearranged” responses aimed to the to-be-forgotten old pairs with those aimed to the to-be-remembered rearranged pairs, which actually represented the complete failure of associative retrieval. Therefore, item retrieval and associative retrieval were indexed by FN400 and LPC respectively, with associative retrieval more inhibited than item retrieval.

## Introduction

Episode memory consists of item memory, which refers to memory for item information, and associative memory, which refers to memory for associative information between single item and other item (or spatial-temporal characteristics of its own). Taking word pairs for example, item information comes from individual words of a word pair, and associative information concerns the relationship or link between the two words. Increasing evidence coming from cognitive psychology (Cleary et al., 2001; Westerman, 2001), neuropsychology (Mayes et al., 2001, 2002) and neuroimaging studies (Lepage et al., 2003; Achim and Lepage, 2005) has indicated that associative memory is different from item memory. However, evidence supporting dissociation from the perspective of time process is inadequate.

The dual process model posits that recognition memory is subdivided into two functionally and neurally separable components: recollection and familiarity (Yonelinas, 2002). Recollection is the retrieval of spatiotemporal context or other details associated with a previously experienced event. In contrast, familiarity is a kind of feeling that the event is previously experienced without recalling of relevant details. They are indexed by different ERP components, i.e., FN400 (mid-frontal old/new effect) and LPC (late positive complex) respectively (Curran and Hancock, 2007; Diana et al., 2007; Addante et al., 2012). FN400 is a negative-going ERP deflection in the time window of 300–500 ms and is maximum at frontal electrodes. LPC is a positive shift potential from 500 to 800 ms and is maximum at parietal sites. For both of the FN400 and LPC, the amplitude is more positive for old items relative to new items (Rugg and Curran, 2007).

If there is a one-to-one mapping relationship both between item retrieval and familiarity and between associative retrieval and recollection, then it can be inferred that item retrieval occurred earlier than associative retrieval. Although the one-to-one mapping relationship was not confirmed by a variety of experiments using a response deadline procedure (Rotello and Heit, 2000; Jones, 2005), an experiment conducted by Lyu et al. (2015) in our laboratory provided direct evidence. They found rearranged pairs elicited more positive potentials than new pairs, while the ERP difference between old and rearranged pairs did not reach significance in 200–400 ms interval. On the contrary, old pairs elicited more positive potentials than rearranged pairs, while there was no significant difference between rearranged pairs and new pairs in 400–800 ms interval. On the base of a hypothesis that the retrieval of associative information was indexed by significant ERP difference between old and rearranged pairs (Hockley, 1992; Kelley and Wixed, 2001), they inferred that only item information was retrieved in the early window and only associative information was retrieved in the late window. Moreover, Tibon et al. (2014) obtained indirect evidence using unrelated picture pairing. However, Liang and Guo (2012) claimed that item retrieval ran parallel to associative retrieval because they found the waveforms evoked by old pairs diverged from those of rearranged pairs from 200 ms after stimulus onset. In order to eliminate the differences between the results, we are plan to dissociate item and associative retrieval through FN400 and LPC respectively.

Interestingly, several studies suggested that directed forgetting had a selectively impact on recollection and familiarity (Bjork and Bjork, 2003; Racsmány et al., 2008; Van Hooff et al., 2009). Specifically, Van Hooff et al. (2009) reported that old/new effect for remembered to-be-forgotten (TBF) items reached significance in early but reduced in late time window, indicating that “Forget” instruction influenced recollection but not familiarity. Thus, directed forgetting paradigm can be regarded as a new method to dissociate item and associative retrieval. With respect to item method directed forgetting paradigm, participants are presented with a series of stimuli one at a time in the study phase, with some stimuli designated as to-be-remembered (TBR) and others as to-be-forgotten (TBF) by an instruction following each stimulus individually. A directed forgetting (DF) effect is obtained when memory performance for TBF items is inferior to TBR items in the test phase (Bjork, 1972; Bailey and Chapman, 2012; Yang et al., 2012; Brandt et al., 2013). To explain DF effect, the selective rehearsal account emphasizes the distinctive encoding (Bailey and Chapman, 2012; Yang et al., 2012), while the attentional inhibition account emphasizes the important role of attention (Zacks et al., 1996; Nowicka et al., 2009). Recently, the retrieval inhibition account is constructed to emphasize the inhibition in the test phase, which posits that the Forget instruction initiates a process which blocks or inhibits access routes to the representations of successfully encoded TBF stimuli (Nowicka et al., 2009; Van Hooff et al., 2009). Reversed old/new effect reported in their study was regarded an indication of retrieval inhibition.

Traditionally, research on directed forgetting has mainly aimed at item information, but has paid little attention to associative information. Moreover, whether associative information can be directed-forgotten is unsettled. Some researchers reported it was hard to forget associative information (Golding et al., 1994; Hanczakowski et al., 2012). For example, Golding et al. (1994) presented compound word pairs, such as “seat-belt,” and found that participants could not forget the word “belt” when it followed the TBR word “seat.” On the contrary, some researchers found associative information can be directed-forgotten under certain circumstance (Gottlob and Golding, 2007; Bancroft et al., 2013; Hockley et al., 2015). In the study of Bancroft and colleagues, participants were told to generate an association that would relate the two items together following a Remember instruction while not to form any association following a Forget instruction. At test, the participants needed to discriminate between old and rearranged pairs. The authors reported that the recognition memory for association is inferior in TBF condition to in TBR condition. Additionally, none of them investigated the neural correlates of directed forgetting for associative information. Hence, the second goal of the present study is to investigate the neural correlates of directed forgetting for associative information.

In present study, we used associative recognition paradigm combined with directed forgetting paradigm. At study, we asked participants to remember or forget names according to the instruction (item method directed forgetting task). At test, participants were asked to make three kinds of judgments for all studied names regardless of the instruction (old, rearranged, new; associative recognition task). On the basis of previous studies (Nowicka et al., 2009; Van Hooff et al., 2009; Lyu et al., 2015), we proposed the following assumptions. First, item retrieval and associative retrieval are indexed by FN400 at the 300–500 ms interval and LPC at the 500–800 ms interval respectively. Second, both item and associative information can be directed-forgotten, but the neural activity between them is different from each other. Specifically, it is indexed by different ERP effect: DF effect at the 300–500 ms interval vs. significant reversed effect at the 500–800 ms interval.

## Materials and methods

### Participants

Seventeen right-handed students (9 females; aged 19–25 years; mean age = 22.4 years) who are all native Chinese speakers participated in the experiment. One dataset was excluded due to excessive muscle artifacts and electrode drift, leaving 16 for analyses. All the participants have normal or corrected visual acuity, and reported no current or past neurological or psychiatric disease. Each subject signed an informed consent form before experiment and received monetary compensation after experiment. This research was approved by the Human Research Ethics Committee at Capital Normal University, methods were carried out in accordance with its relevant guidelines.

### Materials

**Figure 1:**
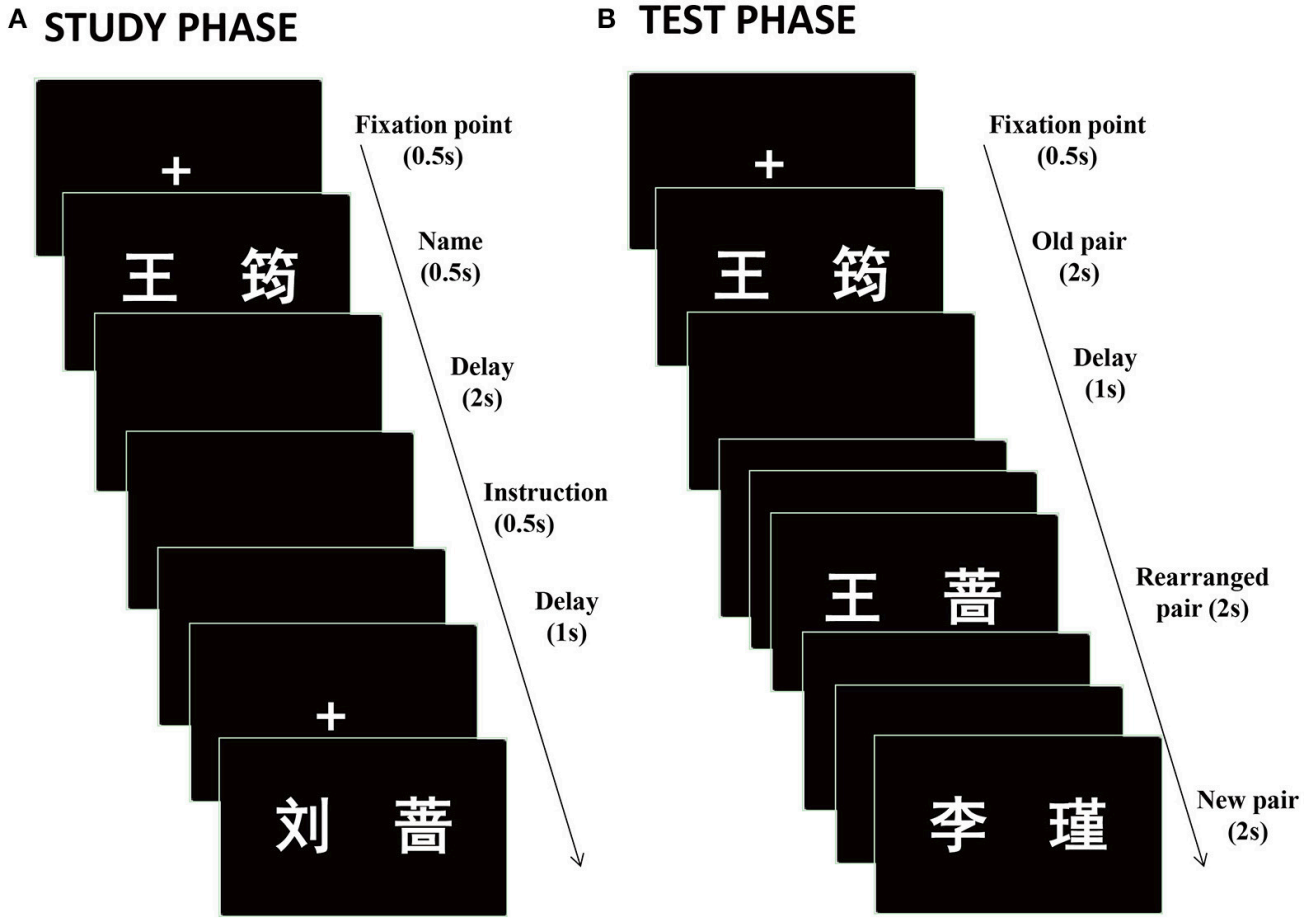
**Schematic representation of the experiment, showing examples of the stimuli. (A)** Study phase, the task was to memory Chinese names according to instruction. **(B)** Test phase, the task was to make three kinds of judgments (“*old,*” “*rearranged*” or “*new*”).

The stimuli consisted of a set of 388 two-character Chinese full names, such as “王筠” (Wang Jun) and “刘蔷” (Liu Qiang) (Figure 1). It should be noted that family name (such as, “Wang” and “Liu”) were selected from a book named “The four hundred most common Chinese family name”, while first name (such as, “Jun” and “Qiang”) were obtained from various Internet sources. For all names, 8 names were used for practice and another 20 names were used for buffer. The remaining 360 names were equally divided into 2 lists: list 1 and list 2, both of which were rearranged as list 3 and list 4, respectively. The left-right study order of the names was also preserved in both old and rearranged pairs. For example, when “王筠” (Wang Jun) and “刘蔷” (Liu Qiang) were presented in list 1, “王蔷” (Wang Qiang) and “刘筠” (Liu Jun) were presented in list 3 (Figure 1). Likewise, when “李瑾” (Li Jin) and “张娟” (Zhang Juan) were presented in list 2, “李娟” (Li Juan) and “张瑾” (Zhang Jin) were presented in list 4. In test phase, for half of participants, list 1, list 3 and list 2 were used as old, rearranged and new stimuli respectively (eg., “王筠”, “王蔷”, “李瑾”), whereas for the other halves, list 2, list 4 and list 1 were used as old, rearranged and new stimuli respectively (eg., “李瑾”, “李瑾”, “王筠”). The test order of old and rearranged trials was controlled by ABBA balance method. Eleven students who did not take part in the formal experiment rated the familiarity of the names on a 5-point Likert scale ranging from 1 = lowest familiarity to 5 = highest familiarity. According to the statistical analysis of rating results, the four lists equaled in terms of familiarity (mean: list 1 = 2.57, list 2 = 2.51, list 3 = 2.63, list 4 = 2.48; *F*(3, 30) = 1.29, *p* > 0.05).

### Procedure

The experiment included a study phase and a test phase (Figure 1). In the study phase, participants firstly received a practice before formal experiment. They were informed that they needed remember or forget names according to instruction. Formal study consisted of 5 blocks, each of which contained 44 names bounded by filler names (two primacy buffers and two recency buffers). For the remaining 40 names, 20 names were instructed to remember, the other 20 names were instructed to forget. Each study trial initiated by a 500 ms presentation of a fixation cross, and then followed by a 500 ms presentation of a name. After names, a blank screen lasted for 2000 ms was inserted. Then, an instruction “记住” (denotes Remember) or “忘记” (denotes Forget) was displayed for 500 ms. Finally, another blank screen was shown for 1000 ms. The order of trials was pseudo-random with the constraint of no more than three consecutive trials for each type of instruction appearing in sequence. All stimuli were presented in white against a black background. After the study phase, participants had a rest of 2 min.

During the test phase, participants also firstly received practice in order to adapt to the task demanding. Formal test consisted of 5 blocks, each with 108 names (36 old, 36 rearranged, 36 new). Each test trial began with a fixation cross lasting for 500 ms, then a name (visual angle 5.30° × 1.43°) was presented for 2000 ms followed by a 1000 ms blank screen (Figure 1). Participants had to make a response before the next fixation cross presented. If both family name and first name in pairs were studied and encoded together at study, they would be judged as “*old*”; if both of them were studied, but not encoded together at study, they would be judged as “*rearranged*”; if both of them were not studied at all, they would be judged as “*new*.” Participants were reminded that they could not make a judgment for old and rearranged pairs according to instruction (“记住” or ”忘记”). The order of trials was also pseudo-random with the constraint of no more than three consecutive trials coming from the same type. Both accuracy and speed of response were emphasized. Response buttons for “*old*” and “*new*” were counterbalanced across participants.

### ERP recording and analyses

Electroencephalographic (EEG) data were recorded in test phase of the experiment and measured from 62 Ag/AgCl electrodes embedded in an elastic cap with NeuroScan SynAmps system (NeuroScan Inc. Sterling, Virginia, USA). The electrode locations adhered to the extended international 10–20 system. All channels were referenced to left mastoid on-line and re-referenced off-line to averaged mastoids. EEG and EOG were amplified using a 0.01–100 Hz band pass and sampled at 500 Hz. Impedance was kept below 5 kΩ. Data were band pass filtered from 0.05 to 40 Hz off-line. EOG blink artifacts were corrected using a linear regression estimate. The averaged epoch was 1200 ms, including 200 ms prior to stimulus onset. Baseline corrections were performed using mean amplitudes of pre-stimulus onset. Trials exceeding ±90 μV were rejected.

We analyzed all data with SPSS 21.0. Repeated-measures ANOVA adjusted by Greenhouse-Geisser corrections when assumptions of sphericity in the repeated measures analyses were violated. The alpha level was 0.05. The analysis of behavioral data focused on accuracy and reaction times (RTs). The ERP amplitudes were averaged at two latency intervals in the test phase (300–500 ms and 500–800 ms) and over sets of midline electrode clusters along the anterior-posterior axis (frontal: F3/Fz/F4; central: C3/Cz/C4; parietal: P3/Pz/P4), which were selected on the basis of previous studies (Van Hooff et al., 2009; Tibon et al., 2014). The ERP amplitude for a certain electrode site was the average of three selected electrodes, for instance, the ERP amplitude for frontal area was the average of F3, Fz, and F4. Likewise, the ERP amplitude for a certain hemisphere was the average of three selected electrodes, for instance, the ERP amplitude for left hemisphere was the average of F3, C3, and P3.

This experiment constituted a 2 (Instruction: remember vs. forget) × 3 (Stimulus Type: old vs. rearranged vs. new) × 3 (Response Type: old vs. rearranged vs. new) incomplete within-subjects design. This design was necessarily incomplete because the instruction manipulation was not varied for new stimulus. Thus, we sorted the EEG data into 15 experimental conditions to examine the event-related potentials elicited by the names. For old names in TBR condition, if they were correctly remembered and judged by participants as “old”—TBR_R; if they were forgotten and were judged as “new”—TBR_F; finally, they could be judged as “rearranged”—TBR_r. For old names in TBF condition, if they were remembered and judged by participants as “old”—TBF_R, if they were forgotten and were judged as “new”—TBF_F; finally, they could be judged as “rearranged”—TBF_r. For rearranged names which consisted of items from names following by the same Remember or Forget instruction, they were denoted as TBRr and TBFr respectively. They could be judged as “old” (TBRr_R, TBFr_R), “new” (TBRr_F, TBFr_F) or “rearranged” (TBRr_r, TBFr_r). For new names, they could be judged as “old” (N_R), “new” (correctly rejected; N_CR) or “rearranged” (N_r). All abbreviations for each condition were shown in Table 1.

**Table 1:** **Abbreviations for each condition**.

**Stimulus type**	**Instruction**	**Response type**		
		**Old**	**Rearranged**	**New**
Old	Remember	TBR_R	TBR_r	TBR_F
	Forget	TBF_R	TBF_r	TBF_F
Rearranged	Remember	TBRr_R	TBRr_r	TBRr_F
	Forget	TBFr_R	TBFr_r	TBFr_F
New		N_R	N_r	N_CR

According to our purpose, we selectively focused on 6 conditions: TBR_R, TBF_R, TBF_r, TBRr_r, TBFr_r, and N_CR condition. The EEG of the selected 6 conditions were separately overlapped and averaged. The mean numbers (standard deviation) of artifact-free trials for them were as follows: TBR_R 61 (10), TBF_R 26 (9), TBF_r 33 (10), TBRr_r 39 (7), TBFr_r 29 (6), and N_CR 130 (25). The mean trial numbers (standard deviation) of the rest 9 conditions were as fol1ows: TBR_F 7 (5), TBR_r 19 (6), TBF_F 29 (12), TBRr_R 13 (5), TBRr_F 7 (5), TBFr_R 10 (5), TBFr_F 19 (6), N_R 9 (7), N_r 35 (20). There were two main reasons why we abandoned these conditions: firstly, the correlation of most conditions with our experiment purpose was low; secondly, trial numbers of these conditions were not sufficient to support further analysis (less than 16).

The analysis of ERP results was conducted including the following three steps. First, in order to investigate the dissociation between item retrieval and associative retrieval, we examined overall differences among TBR_R, TBRr_r, and N_CR condition. Both the stimuli and responses in these conditions differed from each other, but participants made correct responses in each condition. In Lyu et al. (2015) and Tibon et al. (2014), associative recognition was indexed by activation for old vs. rearranged pairs (i.e., a less negative deflection in response to old pairs relative to rearranged pairs). Item recognition was indexed by activation for old/rearranged pairs vs. new pairs, (i.e., greater negativity for new pairs compared with old and rearranged pairs). In this study, associative retrieval was defined by the ERP difference between TBR_R and TBRr_r condition. Item retrieval was defined by the ERP difference between TBR_R/TBRr_r, and N_CR condition.

Second, in order to investigate the DF effect, we examined overall differences among TBR_R, TBF_R, and N_CR condition. In Nowicka et al. (2009) and Van Hooff et al. (2009), directed forgetting for single items was defined by a more negative deflection in response to remembered TBF item relative to remembered TBR item. Meanwhile, they did not find difference between remembered TBF item and new item. In this study, DF effect was defined by the ERP difference both between TBF_R and TBR_R condition and between TBF_R and N_CR condition. As both TBF_R and TBR_R condition contained item and associative information, the ERP difference only reflected some uncertain kind of information was directed-forgotten (item, associative information, or both).

Third, we examined the DF effect for associative information. In Nowicka et al. (2009) and Van Hooff et al. (2009), directed forgetting for single items was also defined by a more negative deflection in response to forgotten TBF item relative to remembered TBF item and new item. In this study, we conducted three contrasts concerning associative information by using the similar logic to the “reversed old/new effect” in item recognition: (1) TBF_r vs. TBF_R condition. Participants in TBF_r condition correctly hit item information but wrongly missed associative information in the pair while they both correctly hit item and associative information in TBF_R condition. Therefore, the ERP difference between TBF_r and TBF_R condition reflected pure associative retrieval. Here, DF effect for associative information can be defined by a more negative deflection in response to TBF_r relative to TBF_R condition. (2) TBF_r vs. TBFr_r condition. Participants in TBFr_r condition not only correctly hit item information but also correctly rejected associative information in the pair. Therefore, the ERP difference between TBF_r and TBFr_r condition also reflected pure associative retrieval. DF effect for associative information can also be indexed by a more negative deflection in response to TBF_r relative to TBFr_r condition. (3) TBF_r vs. TBRr_r condition. Participants in TBRr_r condition not only correctly hit item information but also correctly rejected associative information in the pair. Although participants made correct responses to item information in TBF_r and TBRr_r conditions, the context of responses was different: to-be-forgotten in TBF_r condition while to-be-remembered in TBRr_r condition. Thus, the ERP difference between TBF_r and TBRr_r condition partly reflected mental process with respect to item information. However, they also partly reflected directed forgetting for associative information (i.e., a less positivity for TBF_r compared with TBRr_r condition).

In addition, to exclude the possibility that the between-condition difference in the 500–800 ms time window may be correlated with the difference in the 300–500 ms time window, we planned to confirm the independency between the two time windows by dissociating the two ERP components (i.e., FN400 and LPC). According to previous studies (Curran and Hancock, 2007), we determined to conduct topographical analyses for old/new effects. If the interaction of time window × location was significant, we can refer that the neuronal sources concerning the two components were different.

## Results

### Behavioral data

**Figure 2:**
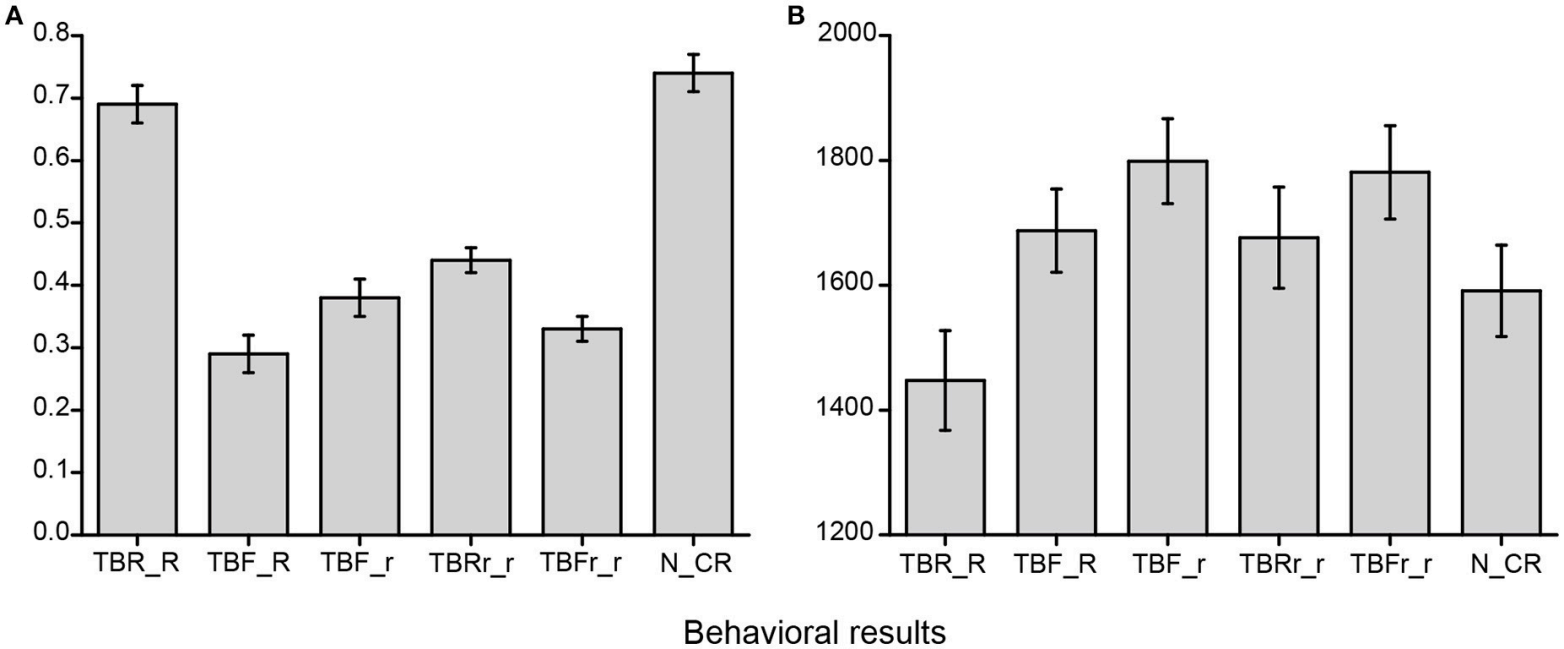
**Mean accuracy and Reaction times (RTs) in each condition. (A)** Mean accuracy for selected conditions. **(B)** Mean RTs for selected conditions.

The mean accuracy and RTs in each condition are presented in Table 2 and Figure 2. According to our goal, we conducted planned comparison for these selected conditions. Because the following five contrasts between different conditions in accuracy and RTs were independent on each other, we did not perform correction for the multiple comparisons.

**Table 2:** **Mean accuracy and Reaction times (RTs) in each condition (standard error of the mean)**.

	**Accuracy**		**RTs**	
	**Mean**	***SE***	**Mean**	***SE***
TBR_R	0.69	0.03	1447.46	80.03
TBF_R	0.29	0.03	1687.23	66.78
TBF_r	0.38	0.03	1798.48	67.99
TBRr_r	0.44	0.02	1675.97	80.99
TBFr_r	0.33	0.02	1780.73	74.56
N_CR	0.74	0.03	1591.12	73.09

For mean accuracy, paired *t*-test showed that mean accuracy in TBR_R condition was greater than TBRr_r condition [*t*_(15)_ = 10.406, *p* < 0.001]. Mean accuracy in TBF_R condition was lower than TBR_R condition [*t*_(15)_ = −10.613, *p* < 0.001]. Mean accuracy in TBF_r condition was greater than TBF_R condition [*t*_(15)_ = 2.121, *p* = 0.05]. Mean accuracy in TBF_r condition was greater than TBFr_r condition [*t*_(15)_ = 2.535, *p* = 0.023]. Mean accuracy in TBF_r condition was lower than TBRr_r condition [*t*_(15)_ = −2.869, *p* = 0.012].

For RTs, paired *t*-test showed that RTs in TBR_R condition was shorter than TBRr_r condition [*t*_(15)_ = −7.766, *p* < 0.001]. RTs in TBF_R condition was shorter than TBR_R condition [*t*_(15)_ = −6.379, *p* < 0.001]. RTs in TBF_r condition was longer than TBF_R condition [*t*_(15)_ = 4.138, *p* = 0.001]. RTs in TBF_r condition was not shorter than TBFr_r condition [*t*_(15)_ = 0.737, *p* = 0.473]. RTs in TBF_r condition was longer than TBRr_r condition [*t*_(15)_ = 2.857, *p* = 0.012].

Participants in TBR_R condition made response more accurately and quickly than in TBRr_r condition, which indicated identifying rearranged stimuli was a difficult task. Overall, performance in TBF_R condition was inferior to that in TBR_R condition, indicating that DF effect was present. Associative information may be directed-forgotten by comparing TBF_r condition with TBF_R, TBFr_r, and TBRr_r condition.

### ERP data

#### The dissociation between item retrieval and associative retrieval

**Figure 3:**
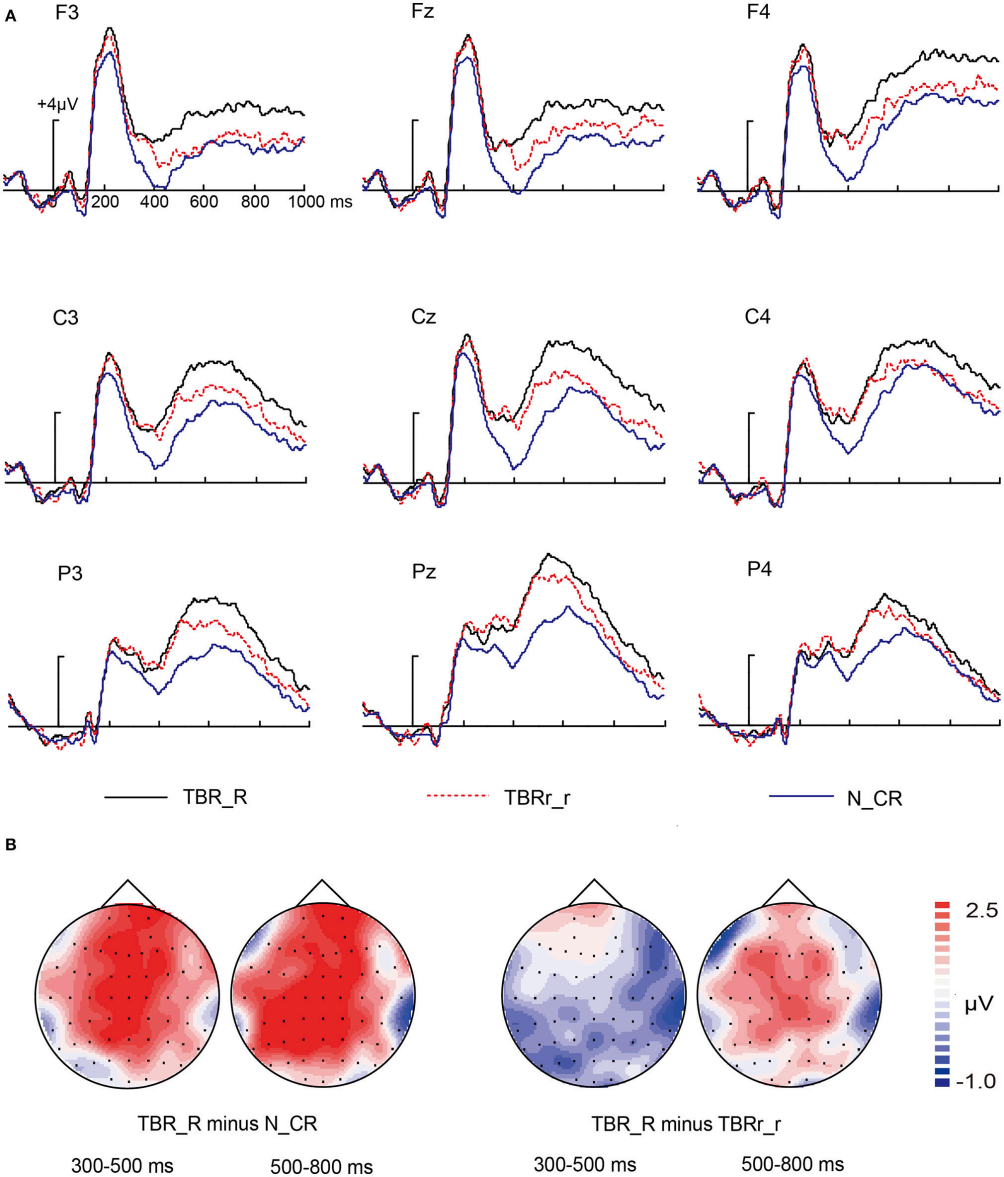
**ERP for TBR_R, TBRr_r, and N_CR condition. (A)** Waveforms are shown from F3, Fz, F4, C3, Cz, C4, P3, Pz, and P4 electrodes. **(B)** The left two topographical plots depict ERP differences between TBR_R condition and N_CR condition at 300–500 ms interval and 500–800 ms interval; the right two topographical plots depict ERP differences between TBR_R condition and TBRr_r condition at 300–500 ms interval and 500–800 ms interval.

The neural activity elicited by TBR_R condition, TBRr_r condition, and N_CR condition are shown in Figure 3. The visual observation of Figure 3 showed that the waveforms diverged around 300 ms after stimulus onset. TBR_R condition evoked more positive activity than N_CR condition. TBRr_r condition also evoked more positive activity than N_CR condition. However, the difference between TBR_R condition and TBRr_r condition did not reach significance before 500 ms. A 3 × 3 × 3 repeated measures MANOVA was performed with the following factors: “condition” (3 levels: TBR_R, TBRr_r, N_CR), “location” (3 levels: frontal, central, parietal) and “laterality” (3 levels: left, middle, right).

For 300–500 ms interval, the main effect of condition was significant [*F*_(2, 30)_ = 17.095, *p* < 0.001]. *Post-hoc* comparison showed that N_CR condition was more negative than TBR_R condition and TBRr_r condition (both *p* < 0.001), whereas no difference was observed between TBR_R condition and TBRr_r condition (*p* = 0.487). The reliability of the null hypothesis was confirmed with Bayesian factor analysis (Rouder et al., 2009, 2012), which indicated that the null hypothesis (no difference in amplitude between the TBR_R and TBRr_r condition) was 7.24 times more likely to be true than the alternative hypothesis (a difference in amplitude between the TBR_R and TBRr_r condition). Although the condition × location × laterality interaction was insignificant [*F*_(8, 120)_ = 1.512, *p* = 0.196], the condition × location and condition × laterality interaction were significant [*F*_(4, 60)_ = 3.215, *p* = 0.044; *F*_(4, 60)_ = 4.835, *p* = 0.006, respectively]. In order to dissolve the interaction involving the factors “location” and “laterality,” separate ANOVA with the factor “condition” was performed for each electrode site. Significant effects of condition were obtained for electrodes F3, Fz, F4, C3, Cz, and C4 (all *p* < 0.001). Subsequent *post-hoc* comparison obtained similar modulation for each electrode except for the magnitude of *p* value. Specifically, N_CR condition was more negative than TBR_R condition for electrodes F3, Fz, F4, C3, Cz, and C4 (all *p* < 0.001). N_CR condition was more negative than TBRr_r condition for electrodes F3 (*p* = 0.008), Fz (*p* = 0.007), F4 (*p* = 0.004), C3 (*p* = 0.001), Cz (*p* < 0.001), and C4 (*p* = 0.03). No difference was observed between TBR_R condition and TBRr_r condition for electrodes F3 (*p* = 0.103), Fz (*p* = 0.067), F4 (*p* = 0.276), C3 (*p* = 0.57), Cz (*p* = 0.388), and C4 (*p* = 0.726).

For 500–800 ms interval, the main effect of condition was significant [*F*_(2, 30)_ = 8.423, *p* = 0.001]. *Post-hoc* comparison revealed that more positive amplitude was found for TBR_R condition relative to N_CR condition (*p* < 0.001), but TBRr_r condition was not more positive than N_CR condition (*p* = 0.141). Critically, TBR_R condition was more positive than TBRr_r condition (*p* = 0.032). The condition × location, condition × laterality and condition × location × laterality interaction were insignificant [*F*_(4, 60)_ = 0.734, *p* = 0.513; *F*_(4, 60)_ = 1.912, *p* = 0.134; *F*_(8, 120)_ = 1.965, *p* = 0.094, respectively].

#### Directed forgetting effect for the whole name

**Figure 4:**
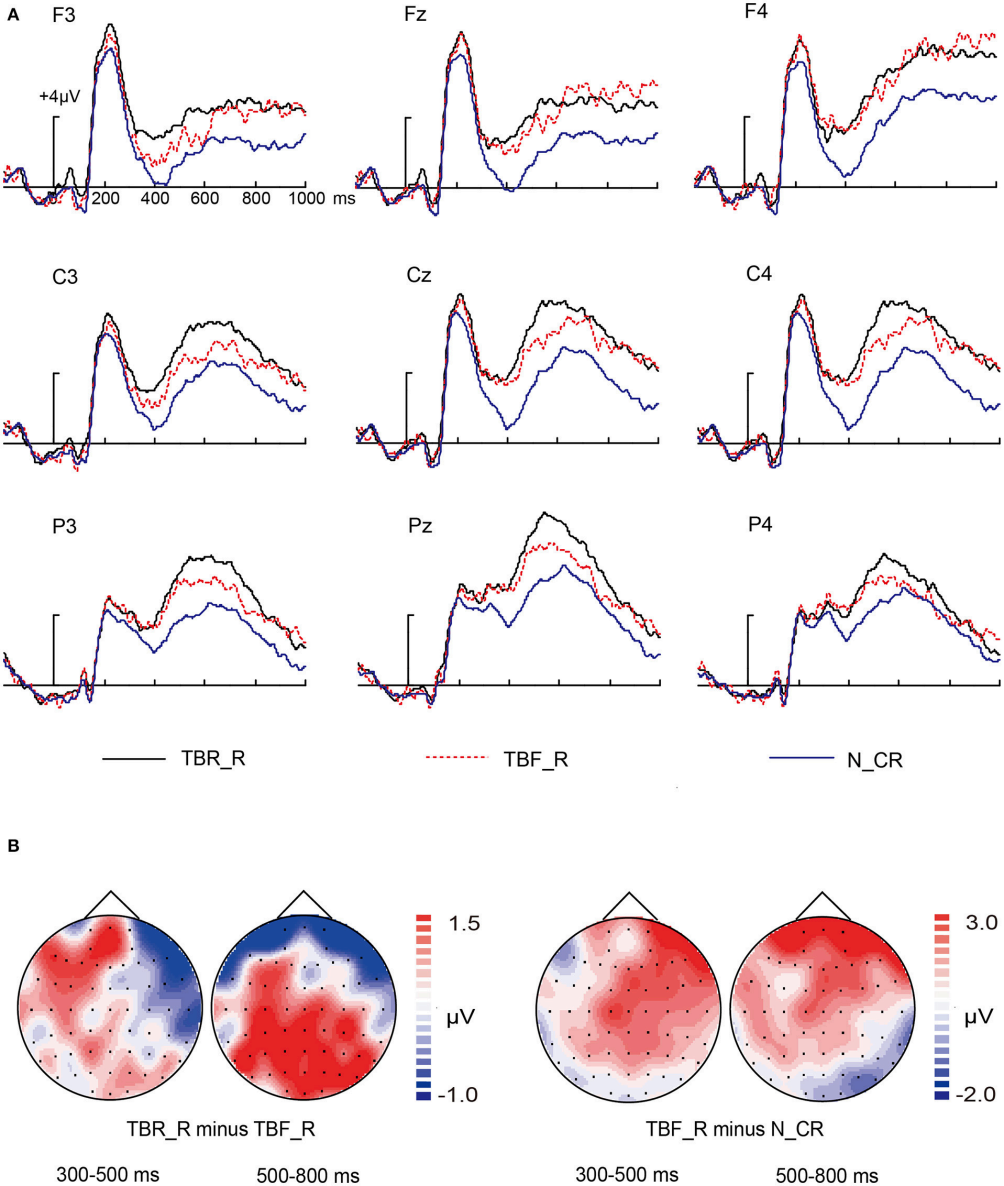
**ERP for TBF_R, TBR_R, and N_CR condition. (A)** Waveforms are shown from F3, Fz, F4, C3, Cz, C4, P3, Pz, and P4 electrodes. **(B)** The left two topographical plots depict ERP differences between TBF_R condition and TBR_R condition at 300–500 ms interval and 500–800 ms interval; the right two topographical plots depict ERP differences between TBF_R condition and N_CR condition at 300–500 ms interval and 500–800 ms interval.

The waves provoked by TBR_R, TBF_R, and N_CR condition are exhibited in Figure 4. The visual observation of Figure 4 showed that the waveforms of TBF_R condition were more negative activity than that of TBR_R condition from 300 to 800 ms. TBF_R condition seemed to evoke more positive activity than N_CR condition. We conducted a 3 × 3 × 3 repeated measures MANOVA with the factors of “condition” (3 levels: TBR_R, TBF_R, N_CR), “location” (3 levels: frontal, central, parietal) and “laterality” (3 levels: left, middle, right) at intervals of 300–500 ms and 500–800 ms.

For 300–500 ms interval, the main effect of condition was significant [*F*_(2, 30)_ = 23.135, *p* < 0.001]. *Post-hoc* comparison showed that N_CR condition was more negative than TBR_R condition and TBF_R condition (both *p* < 0.001), whereas no difference was observed between TBR_R condition and TBF_R condition (*p* = 0.082). Although the condition × location interaction was insignificant [*F*_(4, 60)_ = 0.534, *p* = 0.582], the condition × laterality and condition × location × laterality interaction were significant [*F*_(4, 60)_ = 7.562, *p* < 0.001; *F*_(8, 120)_ = 2.486, *p* = 0.039]. Dissolving the interactions involving the factors of location and laterality, separate condition ANOVA was calculated for each electrode site yielding significant effects of condition for all electrodes (all *p* < 0.001). Subsequent *post-hoc* comparison showed that ERP for TBF_R condition did not differ from TBR_R condition for all electrodes (all *p* > 0.05), except for electrode F3 (*p* = 0.033). ERP for N_CR were more negative-going than ERP for TBF_R condition for electrodes Fz (*p* = 0.003), F4 (*p* = 0.001), C3 (*p* = 0.045), Cz (*p* = 0.001), C4 (*p* = 0.001), P3 (*p* = 0.011), Pz (*p* = 0.003), and P4 (*p* = 0.011).

For 500–800 ms interval, the main effect of condition was significant [*F*_(2, 30)_ = 7.956, *p* = 0.002]. *Post-hoc* comparison showed that TBR_R condition and TBF_R condition were more positive than N_CR condition (*p* < 0.001, *p* = 0.035, respectively), whereas no difference was observed between TBR_R condition and TBF_R condition (*p* = 0.167). Although the condition × location and condition × laterality interaction were insignificant [*F*_(4, 60)_ = 1.261, *p* = 0.299; *F*_(4, 60)_ = 1.877, *p* = 0.161, respectively], the condition × location × laterality interaction was significant [*F*_(8, 120)_ = 2.422, *p* = 0.048]. To dissolve the three-way interaction, separate ANOVA with the factor “condition” was conducted for each electrode site yielding significant effects of condition for all electrodes (all *p* < 0.05). Subsequent *post-hoc* comparison revealed that TBF_R condition elicited more negative-going waves than TBR_R condition for electrodes P3 (*p* = 0.027) and Pz (*p* = 0.041). However, ERP for TBF_R were more positive-going than ERP for N_CR condition for electrodes Fz (*p* = 0.016), F4 (*p* = 0.013), Cz (*p* = 0.043), and P3 (*p* = 0.037).

#### Directed forgetting for associative information

**Figure 5:**
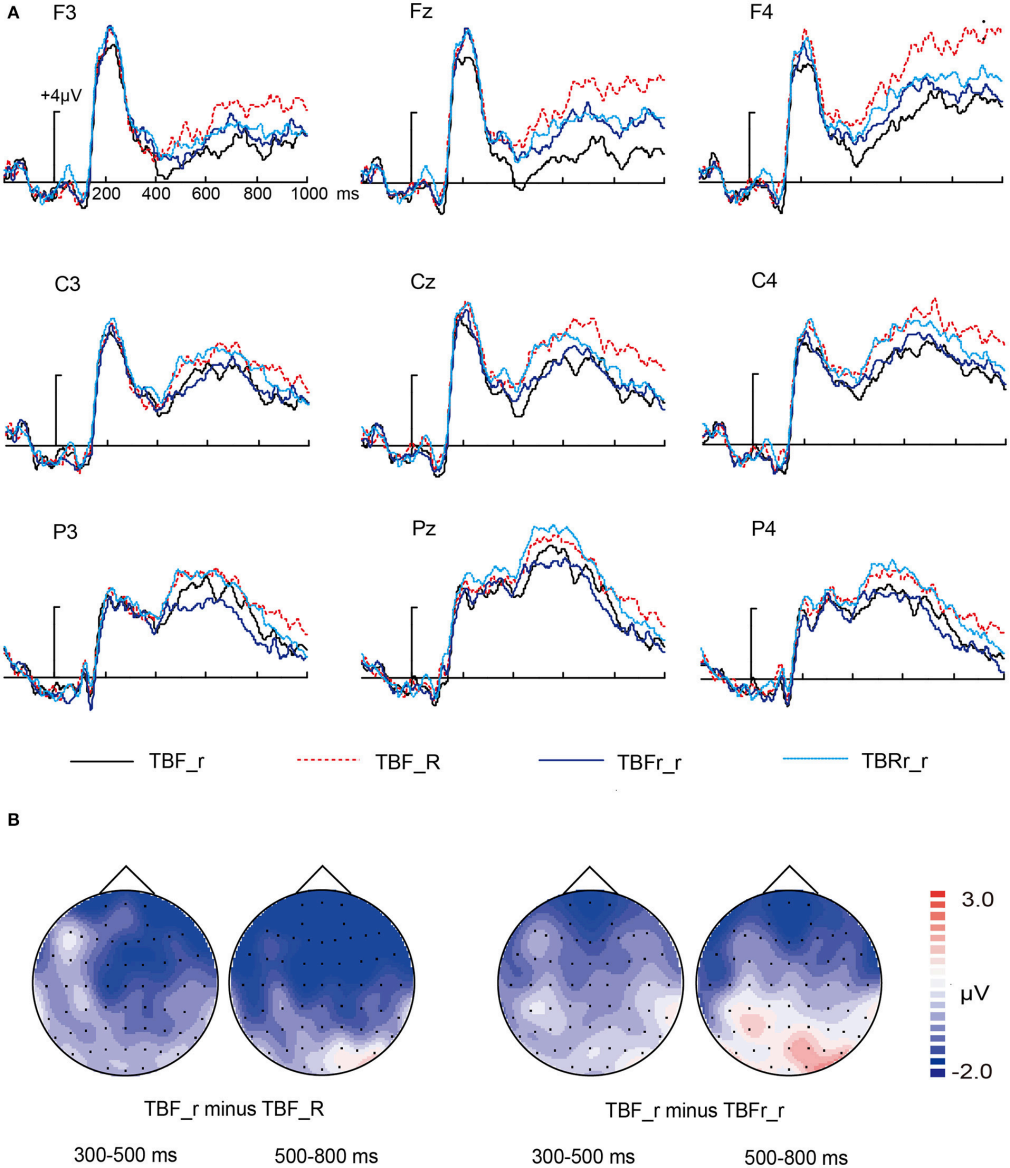
**ERP for TBF_r, TBF_R, TBFr_r, and TBRr_r condition. (A)** Waveforms are shown from F3, Fz, F4, C3, Cz, C4, P3, Pz, and P4 electrodes. **(B)** The left two topographical plot depict ERP differences between TBF_r condition and TBF_R condition at 500–800 ms interval; the right two topographical plots depict ERP differences between TBF_R condition and TBFr_r condition at 300–500 ms interval and 500–800 ms interval.

The neural activity elicited by TBF_r, TBF_R, TBFr_r, and TBRr_r condition are shown in Figure 5. In general, the visual observation of Figure 5 showed that TBF_r condition elicited more negative amplitude than TBF_R, TBFr_r, and TBRr_r condition at two selected time intervals. In order to determine when and where the reversed effect occurred, we conducted the following repeated measures MANOVA.

First, a 2 × 3 × 3 repeated measures MANOVA was performed with the following factors: “condition” (2 levels: TBF_r, TBF_R), “location” (3 levels: frontal, central, parietal) and “laterality” (3 levels: left, middle, right).

For 300–500 ms interval, the main effect of condition was significant, ERP for TBF_r condition were more negative than ERP for TBF_R condition [*F*_(1, 15)_ = 4.544, *p* = 0.05]. Both the two-way condition × location and three-way interaction were insignificant [*F*_(2, 30)_ = 1.083, *p* = 0.332; *F*_(4, 60)_ = 1.779, *p* = 0.159, respectively]. However, the condition × laterality interaction was significant [*F*_(2, 30)_ = 5.766, *p* = 0.008]. To dissolve the two-way interaction, separate ANOVA with the factor “condition” was conducted for each electrode site yielding significant effects of condition for electrodes Fz (*p* = 0.029), F4 (*p* = 0.018), Cz (*p* = 0.01), and C4 (*p* = 0.039). For 500–800 ms interval, the main effect of condition was significant, ERP for TBF_r condition were more negative than ERP for TBF_R condition [*F*_(1, 15)_ = 6.016, *p* = 0.027]. Although the condition × laterality and condition × location × laterality interaction were insignificant [*F*_(2, 30)_ = 2.787, *p* = 0.092; *F*_(4, 60)_ = 1.388, *p* = 0.254], the condition × location interaction was significant [*F*_(2, 30)_ = 6.102, *p* = 0.01]. To dissolve the two-way interaction, separate ANOVA with the factor “condition” was conducted for each electrode site yielding significant effects of condition for electrodes Fz (*p* = 0.006), F4 (*p* = 0.002), and Cz (*p* = 0.039).

Second, a 2 × 3 × 3 repeated measures MANOVA was performed with the following factors: “condition” (2 levels: TBF_r, TBFr_r), “location” (3 levels: frontal, central, parietal) and “laterality” (3 levels: left, middle, right).

For 300–500 ms interval, the main effect of condition was insignificant, that is, ERP for TBF_r condition did not differ from ERP for TBFr_r condition [*F*_(1, 15)_ = 0.716, *p* = 0.411]. The null hypothesis was also confirmed with Bayesian factor analysis (Bayesian factor = 5.35). All interactions concerning “condition” were insignificant: the condition × location interaction [*F*_(2, 30)_ = 2.687, *p* = 0.097], the condition × laterality interaction [*F*_(2, 30)_ = 1.498, *p* = 0.241], and the condition × location × laterality interaction [*F*_(4, 60)_ = 0.18, *p* = 0.916]. For 500–800 ms interval, the main effect of condition was insignificant, that is, ERP for TBF_r condition did not differ from ERP for TBFr_r condition [*F*_(1, 15)_ = 0.155, *p* = 0.699]. The null hypothesis was also confirmed with Bayesian factor analysis (Bayesian factor = 7.76). Although the condition × laterality and condition × location × laterality interaction were insignificant [*F*_(2, 30)_ = 1.858, *p* = 0.18; *F*_(4, 60)_ = 0.689, *p* = 0.564, respectively], the condition × location interaction was significant [*F*_(2, 30)_ = 8.193, *p* = 0.003]. To dissolve the two-way interaction, separate ANOVA with the factor “condition” was conducted for each electrode site yielding significant effects of condition only for electrode Fz (*p* = 0.017).

Third, a 2 × 3 × 3 repeated measures MANOVA was performed with the following factors: “condition” (2 levels: TBF_r, TBRr_r), “location” (3 levels: frontal, central, parietal) and “laterality” (3 levels: left, middle, right).

For 300–500 ms interval, the main effect of condition was significant, that is, ERP for TBF_r condition were more negative-going than ERP for TBRr_r condition [*F*_(1, 15)_ = 5.754, *p* = 0.03]. All interactions with “condition” did not reach significance: the condition × location interaction [*F*_(2, 30)_ = 0.356, *p* = 0.644], the condition × laterality interaction [*F*_(2, 30)_ = 2.599, *p* = 0.106], and the condition × location × laterality interaction [*F*_(4, 60)_ = 0.091, *p* = 0.956]. For 500–800 ms interval, the main effect of condition was significant, that is, ERP for TBF_r condition were more negative-going than ERP for TBRr_r condition [*F*_(1, 15)_ = 4.578, *p* = 0.049]. The condition × location, condition × laterality, and condition × location × laterality interaction were insignificant [*F*_(2, 30)_ = 0.489, *p* = 0.548; *F*_(2, 30)_ = 1.78, *p* = 0.195; *F*_(4, 60)_ = 0.532, *p* = 0.665, respectively].

#### Topographic analyses

To dissociate the two ERP components (i.e., FN400 and LPC), topographical analyses for old/new effects were performed on the old and new differences using rescaled data with the vector normalization approach (McCarthy and Wood, 1985). As indicated by Figure 3B, old/new effects for the two intervals exhibited different topographical distributions. We made topographic comparison using averaged amplitude values from all scalp electrodes after overall amplitude differences were removed. Specifically, we compared the old/new effects for TBR_R/N_CR condition for 300–500 ms versus 500–800 ms. A repeated measures ANOVA with location (62 electrodes) and time window (early, late) revealed a significant interaction between two factors [*F*_(61, 915)_ = 3.056, *p* = 0.042], demonstrating the observation that the old/new effects tended to be more anterior for 300–500 ms and more posterior for 500–800 ms.

## Discussion

In present study, we employed associative recognition paradigm combined with directed forgetting paradigm to investigate the neural dissociation between item retrieval and associative retrieval. We found three aspects of results related to our purpose. First, old/rearranged effect in TBR condition was found only in 500–800 ms epoch; namely, the two types of episode memory differed in related ERP components: FN400 for item retrieval and LPC for associative retrieval, in line with Lyu et al. (2015). Second, we found that ERP of TBF_r condition was more negative than that of TBF_R, TBFr_r, TBRr_r condition between 500 and 800 ms, which indicated that associative information can be directed-forgotten. This is consistent with the results of Bancroft et al. (2013) and Hockley et al. (2015). Finally, in 300–500 ms interval, TBF_r condition evoked more negative-going potentials than TBRr_r condition (DF effect) but similar to TBFr_r condition (absent DF effect). This can be taken as an indication that item information was directed-forgotten. These different effects probably suggest that neural activity of directed forgetting for item information may be different from that for associative information. This finding is reported in associative recognition study for the first time. The implications of these findings are discussed in the succeeding sections.

### The dissociation between item retrieval and associative retrieval

The behavioral results showed that participants in TBR_R condition were more accurate and faster than in TBRr_r condition, which was consistent with a number of researches on comparing old pairs with rearranged pairs (Donaldson and Rugg, 1998; Liang and Guo, 2012; Lyu et al., 2015). In accordance with the Encoding Specificity Principle (Tulving and Thomson, 1973), item retrieval is more likely to be successful if items are presented at test in the context in which they are studied (old pairing) rather than in a new context (rearranged pairing). In this study, items at test in TBR_R condition appeared in the same context as encoding, whereas items in TBRr_r condition occurred in new context.

For ERP data, it can be inferred that item retrieval and associative retrieval dissociated in different time courses, that is, item retrieval appeared at 300–500 ms interval and associative retrieval appeared at 500–800 ms interval. We found TBR_R condition elicited more positive potentials than N_CR condition both in early and late interval, indicating that some kind of information was retrieved. However, which type of information (item or association) was uncertain. The ERP difference between rearranged and new pairs is thought to reflect item information as both associations in the two type of pairs were not studied in the study phase (Hockley, 1992). Likewise, the ERP difference between old and rearranged pairs is thought to reflect associative information on the base of two hypotheses. One is item information in the two types of pairs has equal extent familiarity (Hockley, 1992), the other one is associative information probably is an all-or-none variable (Kelley and Wixed, 2001). In 300–500 ms interval, the ERP difference between TBR_R condition and TBRr_r condition did not reach significance while TBRr_r condition elicited more positive potentials than N_CR condition, which indicated that participants only retrieved item information in early recognition. In 500–800 ms interval, we obtained a reversal pattern: the ERP difference between TBRr_r condition and N_CR condition was insignificant while TBR_R condition elicited more positive potentials than TBRr_r condition, indicating only associative information was retrieved in late recognition. This pattern of results was consistent with that of Lyu et al. (2015) and Tibon et al. (2014). Tibon et al. (2014) found differences between old and new pairs, and between rearranged and new pairs, but not between old and rearranged pairs by pairwise comparisons in 350–550 ms interval. For the later time window (550–750 ms), this analysis revealed a significant difference between old and rearranged pairs.

The idea that associative retrieval was indexed by the ERP difference between old and rearranged pairs was supported by indirect evidence. Donaldson and Rugg (1998) showed that old and rearranged pairs were associated with different magnitude old/new effects in three time windows (600–900 ms, 900–1200 ms, 1200–1434 ms). They explained that old pairs are more likely to engender recollection of associative information while rearranged pairs may mainly be formed from trials made by default strategy lack of recollection. Similarly, Rugg et al. (1996) found that recognized/recalled items elicited larger old/new effect than those for recognized/unrecalled items from 500 to 800 ms. They attributed this divergence to different source of information: information of recognized/recalled items is in terms of the successful recollection of study episode, whereas information of recognized/unrecalled items bases largely on familiarity. Furthermore, Jäger et al. (2006) found that face elicited old/new effect at 400–700 ms interval in inter-item condition when it was recognized and then forced-choice judged correctly. It suggested that recollection enables retrieving association between arbitrarily different faces.

Item retrieval was demonstrated to occur earlier than associative retrieval. Meanwhile, we provided evidence for the viewpoint that item retrieval relied only on familiarity and associative retrieval relied only on recollection. A lot of researches have revealed that associative retrieval depended only on recollection. Yonelinas (1997) reported that associative recognition is only based on recollection through comparing the ROC curve of item recognition with those of associative recognition. Hockley and Consoli (1999), employing “R/K” paradigm, subsequently found more “remember” response than “know” response in associative recognition. It is generally agreed that item retrieval can be supported by both familiarity and recollection (Yonelinas, 1997; Donaldson and Rugg, 1998; Jäger et al., 2006), but this idea has been challenged by Jacoby (1991) who posited that item retrieval depends only on familiarity. Liang (2013) further posited that there may be more familiar sensation in item retrieval and that recollection may be involved in retrieving the inherent characteristic or contextual details of items. In our experiment, participants made correct judgments via the pronunciation (or spelling) of names without retrieving details like the font, color, position, meaning, and so on. Thus, item retrieval relies merely on familiarity.

### Directed forgetting for associative information and item information

The amplitudes of ERP for TBF_r condition were lower than amplitudes of TBF_R, TBFr_r, TBRr_r condition at 500–800 ms interval (the reversed effect), which was similar to the findings of Nowicka et al. (2009) and Van Hooff et al. (2009). Combined with behavioral results, we concluded that associative information was directed-forgotten.

According to selective rehearsal account, the rote rehearsal for associative information is stopped at the time of presentation of Forget instruction (Bailey and Chapman, 2012; Yang et al., 2012). In other words, Forget instruction results in inhibition of normal encoding processes for TBF associative information. However, some researchers argued that Forget instruction initiates attentional inhibition mechanism, which may withdraw attention from TBF associative information and prevent the return of attention to their representations (Zacks et al., 1996; Nowicka et al., 2009). Both passive stop of rote rehearsal and active inhibition of attention return cause the inferiority of the encoding for associative information of TBF compared to that of TBR. Nevertheless, in accordance with the retrieval inhibitory account (Bjork and Bjork, 2003), the Forget instruction only blocked the access of retrieval for TBF associative information, which is available via successfully encoding but not accessible. In summary, the reversed effect for forgotten TBF names may result from either successful inhibition of encoding processes or retrieval processes or even both.

We found that ERP of TBF_R condition was more negative than that of TBR_R condition at the two selected time window. It can be inferred that the whole name can be directed-forgotten. Unfortunately, we cannot determine which kind of information was directed-forgotten. As directed forgetting for associative information was indexed by three reversed effect at 500–800 ms interval in our experiment, the ERP difference between TBF_R and TBR_R condition at 300–500 ms interval can be taken as an indication of directed forgetting for item information. In addition, the ERP difference between TBF_r condition and TBRr_r condition in 300–500 ms interval reflected the success of item retrieval under Forget instruction. Meanwhile, there was no ERP difference between TBF_r condition and TBFr_r condition in the time window of 300–500 ms. These results in concert reflected that item information was retrieved in the early time window.

In ERP results, the above evidence strongly suggested that remembered item information in TBF context evoked more negative-going potentials relative to that in TBR context. This was in line with two previous studies, which reflected that remembered TBR items are more likely to give rise to a sense of conscious recollection than remembered TBF items (Nowicka et al., 2009; Van Hooff et al., 2009). In behavioral results, we also replicated the critical finding from a body of studies (Paz-Caballero and Menor, 1999; Ullsperger et al., 2000; Nowicka et al., 2009; Van Hooff et al., 2009; Yang et al., 2012) in which participants were more accurate and faster in response to TBR item than to TBF item (i.e., typical DF effect).

### The dissociation between item retrieval and associative retrieval: from the perspective of directed forgetting

The amplitudes of ERP for TBF_R condition were higher than amplitudes of N_CR condition at 300–500 ms interval, that is, remembered item information in TBF context evoked more positive potentials than new item information. This is inconsistent with Nowicka et al. (2009) and Van Hooff et al. (2009). In their study, ERP for remembered TBF items did not differ from that for new items, which was interpreted as inhibition of items in TBF context.

The discrepancy between the studies of Nowicka et al. (2009) and Van Hooff et al. (2009) and the current study might result from the types of stimuli and the encoding tasks. In their study, stimuli were concrete nouns (e.g., “desk,” “news”) which can quickly generate mental imagery corresponding to the referred objects. In contrast, in our experiment, the family name (e.g., “王” “刘”) and first name (e.g., “筠” “蔷”) were both abstract nouns. The concreteness effect of words indicated that concrete words are processed more quickly and accurately, and then better remembered than abstract words (Zhang et al., 2006). Furthermore, there was only one item for each trial in their studies, whereas there were two items in our study, which might aggravate working memory load (Alvarez and Cavanagh, 2004). Finally, for experimental procedure, Van Hooff et al. (2009) inserted a lexical decision task in which all studied words were presented repeatedly between study and test, resulting in deeply process for studied words. In contrast, participants received test immediately in our experiment, which ultimately resulted in weaker memory strength of items. Altogether, memory performance for items in our experiment was worse than theirs, which indicated by different old/new effect.

The amplitudes of ERP for TBF_r condition were lower than amplitudes of TBFr_r condition at 500–800 ms interval, that is, forgotten associative information in TBF context evoked more negative potentials than new associative information. This seemed to indicate that the neural pattern of directed forgetting for associative information differed from that for item information. As already discussed, in our experiment, item retrieval only based on familiarity which is lack of details, but associative retrieval only engendered recollection which contains numerous details. Thus, associative retrieval retrieves more details than item retrieval. In addition, there is a possibility that the degree of inhibition for item retrieval is equal to that for associative retrieval at the very beginning. Because it was acknowledged by Lyu et al. (2015) and our results that item retrieval occurs before associative retrieval and item retrieval has priority for the use of resources. Thus, the resources are adequate for item retrieval so that the implementation of Forget instruction is blocked. However, according to limited resources theory, the remaining resources for associative retrieval are not sufficient for conquering suppression of Forget instruction, accompanied by competition for the same limited resources between the two types of retrieval (Kahneman, 2003). Finally, associative retrieval is more inhibited than item retrieval, which is demonstrated by different ERP effect.

## Conclusion

In summary, the present study focused on resolving the time window wherein dissociation appeared. On the one hand, item retrieval and associative retrieval were dissociated by different ERP components referred to FN400 and LPC respectively. On the other hand, however, item retrieval and associative retrieval were dissociated by different ERP effect referred to DF effect and pronounced reversed old/new effect. Thus, we can conclude that different ERP correlates are associated with item retrieval and associative retrieval. Unfortunately, in the context of current study, it was impossible to determine in which stage inhibition occurred. In order to resolve this problem, future research should aim at encoding phase. Furthermore, it is interesting to investigate whether our results are interacted with the type of association, as between-domain inter-item association is different from within-domain inter-item association used here (Mayes et al., 2007).

## Author contributions

YW designed the experiment, collected data, drawed the figures, and wrote the manuscript. XM, BL, and WW revised the manuscript. CG instructed the whole research.

### Conflict of interest statement

The authors declare that the research was conducted in the absence of any commercial or financial relationships that could be construed as a potential conflict of interest.
